# HOUSEHOLD ENVIRONMENTS AND COGNITIVE DECLINE AMONG MIDDLE-AGED AND OLDER ADULTS IN CHINA

**DOI:** 10.1093/geroni/igad104.3078

**Published:** 2023-12-21

**Authors:** Ye Luo, Dandan Zhao, Xi Pan, Lingling Zhang

**Affiliations:** Clemson University, Clemson, South Carolina, United States; Clemson University, Clemson, South Carolina, United States; Texas State University, San Marcos, Texas, United States; University of Massachusetts Boston, Boston, Massachusetts, United States

## Abstract

Household conditions play a crucial role in the well-being of older adults, who usually spend much longer time in their homes than younger adults. Few studies have examined the compound effects of multiple household conditions on trajectory of cognition function among older adults. This study examines the relationship between household environments and trajectories of cognitive function among middle-aged and older adults in China. It also examines urban/rural, gender, and age variations in this relationship. Using a representative sample of 16,111 respondents aged 45 years and over from four waves of the China Health and Retirement Longitudinal Study (2011-2018), we estimated multi-level linear growth curve models of household social, economic, and physical conditions on cognitive decline over seven years. The results show that older people who lived with spouse but not with children and those with higher living expenditures, better housing quality, and indoor clean fuels for cooking had slower cognitive decline. Living arrangement more strongly predicted men’s cognitive decline while living expenditure, solid fuel use and housing quality significantly predicted only women’s cognitive decline. Only for adults aged 60 year and over, living alone or living with spouse and adult children significantly predicted a faster cognitive decline. Only for rural residents, living alone or living with young children and no adult children significantly predicted a faster cognitive decline. These findings underscore the importance of social policies and programs targeting at improving the living conditions of older adults to help mitigate their cognitive decline.

